# Nerve growth factor is closely related to glucose metabolism, insulin sensitivity and insulin secretion in the second trimester: a case–control study in Chinese

**DOI:** 10.1186/s12986-020-00523-2

**Published:** 2020-11-19

**Authors:** Mengyang Tang, Mingjuan Luo, Wenqian Lu, Rong Zhang, Wei Liang, Jianfen Gu, Xuemei Yu, Xueli Zhang, Cheng Hu

**Affiliations:** 1 The Third School of Clinical Medicine, Southern Medical University, Guangzhou, China; 2 Department of Endocrinology and Metabolism, Fengxian Central Hospital Affiliated to the Southern Medical University, Shanghai, China; 3 Department of Endocrinology and Metabolism, University of Hong Kong Shenzhen Hospital, Shenzhen, China; 4 Shanghai Diabetes Institute, Shanghai Jiao Tong University Affiliated Sixth People’s Hospital, Shanghai, China

**Keywords:** Gestational diabetes, Glucose metabolism, Inflammatory cytokines, Nerve growth factor

## Abstract

**Objective:**

Inflammation-related factors have been shown to play a significant role throughout pregnancy. In this study, we aimed to explore the relationships between selected inflammatory cytokines and gestational diabetes (GDM) in Chinese pregnant women.

**Design and methods:**

This was a 1:1 matched case–control study that included 200 pairs of subjects in the second trimester and 130 pairs of subjects in the third trimester. Serum levels of nerve growth factor (NGF), Interleukin-6 (IL-6), leptin, Interleukin-8 (IL-8), monocyte chemoattractant protein-1 (MCP-1), tumor necrosis factor-alpha (TNF-α) and Interleukin-1beta (IL-1β) were measured by enzyme immunoassay. The associations of these inflammatory factors with metabolic parameters were analysed.

**Results:**

In the second trimester, GDM patients had higher NGF levels and lower IL-8 levels than did normal controls (*P* < 0.001 and *P* = 0.015, respectively). However, in the third trimester, only lower leptin levels were observed in the GDM group (*P* = 0.031). Additionally, in the second trimester, NGF levels were not only positively associated with fasting, 1-h and 2-h glucose levels and the area under curve of glucose, but also positively related to insulin sensitivity and secretion, as suggested by fasting insulin, homeostasis model assessment of insulin resistance (HOMA-IR) and homeostasis model assessment index of β-cell secretion (HOMA-β) (all *P* < 0.05). Moreover, IL-6 and leptin levels were positively correlated with HOMA-IR and HOMA-β, and TNF-α levels were positively related to HOMA-IR (all *P* < 0.05). Except for the relationships between NGF and HOMA-β and TNF-α and HOMA-IR, the other correlations still existed even after adjusting for confounding factors (all *P* < 0.05).

**Conclusion:**

In addition to the positive associations of IL-6 and leptin with insulin resistance and secretion, NGF was higher in the GDM patients and strongly linked to glucose metabolism, insulin resistance and pancreatic β cell function in Chinese pregnant women in the second trimester.

## Background

Gestational diabetes mellitus (GDM) is a pathological state in which pancreatic β cells cannot release adequate insulin to meet the increased insulin demand [1]. Just as type 2 diabetes (T2DM), environmental factors, genetic factors, and epigenetic modifications all contribute to the accelerating GDM epidemic [2, 3]. Currently, chronic insulin resistance, an indirect cause of impaired pancreatic β cell function, is thought to be the major pathological feature of GDM [4–6]. However, explorations of the exact mechanism of insulin resistance in GDM are still limited, with most studies concentrating on T2DM. As one of the leading causes of T2DM, insulin resistance is strongly associated with inflammation [7–10]. In obese patients with T2DM, which feature with macrophage accumulation and chronic inflammation, insulin resistance can be found in many tissues, including adipose tissue, the liver and skeletal muscle [8, 9]. To date, scientists have revealed that one of the central pathogenic mechanisms of insulin resistance is the modulation of inflammation-related factors, which affects the gene expression regulation and metabolism of some lipid-related factors involved in the insulin signalling pathway [11].

Given the close relationship between inflammation and insulin resistance, many studies have focused on the relationships between inflammation-related factors and GDM. A prospective study indicated that increased serum C-reactive protein (CRP) levels could positively predict the morbidity of GDM [12]. Recently, a case–control study from Inner Mongolia also found that there were positive associations of high sensitivity CRP, Interleukin-6 (IL-6), Interleukin-8 (IL-8) and the IL-6/IL-10 ratio with GDM [13]. Other nontypical inflammatory factors such as growth differentiation factor 15 (GDF-15) was also proved to have a close relationship with GDM [14]. These data provide evidence that inflammatory cytokines are important factors in the pathological processes of GDM.

However, studies of the exact relationships between inflammatory factors and some important metabolic indexes in Chinese GDM patients are still limited. Therefore, in this study, through systematic analyses of selected inflammatory cytokines and GDM in both the second and third trimesters, we aimed to explore the differences in serum levels of nerve growth factor (NGF), IL-6, leptin, IL-8, monocyte chemoattractant protein-1 (MCP-1), Interleukin-1 beta (IL-1β) and tumor necrosis factor-alpha (TNF-α) between GDM patients and normal controls, and to analyse the exact correlations of these cytokines with glucose metabolism, insulin resistance and pancreatic β cell function.

## Methods

### Study participants and clinical measurements

All participants and their clinical measurements were described in our previous article [14]. In brief, according to age (± 3 years), pregestational BMI (± 3 kg/m^2^), and gestational week (± 3 weeks), we included 200 GDM patients and 200 matched normal controls in the second trimester as well as 130 GDM patients and 130 matched normal controls in the third trimester. Women with autoimmune disease, thyroid disease, heart trouble, liver or kidney disease, tumors, hematopathy, and other known diseases affecting glucolipid metabolism were excluded from this study. For the GDM groups in the second and third trimesters, there were no significant differences in age or pregestational BMI, and the same was true for the normal glucose tolerance (NGT) groups (both *P* > 0.05). This study was conformed to the provision of the Declaration of Helsinki and approved by The Medical Ethics Committee of University of Hong Kong Shenzhen Hospital. We have obtained written informed consent from every participant before undertaking this study.

### Measurement of serum inflammatory cytokines

NGF, IL-6, leptin, IL-8, MCP-1, TNF-α and IL-1β were detected using a MILLIPLEX® MAP kit (Merck Corp., New Jersey, USA) on a MAGPIX analyser (Merck Corp.). This assay has been proven to be highly sensitive for all inflammatory cytokines, with minimum detectable doses of human NGF, IL-6, leptin, IL-8, MCP-1, TNF-α and IL-1β of 0.3, 0.2, 3.8, 19, 1.2, 0.3 and 0.4 pg/ml, respectively. No or negligible cross-reactivity of these antibodies across the seven inflammatory cytokines was detected. Moreover, except for the inter-assay variation of TNF-α, which was less than 20%, the intra-assay variations and inter-assay variations for all of these cytokines were less than 10% and less than 15%, respectively.

### Statistical analysis

SAS (version 8.0) was used to perform statistical analyses. GraphPad Prism software (version 7.0) was used to plot the graphs. All of the data were subjected to a normality test. If the data were normally distributed, they were expressed as the mean ± SD. Data with an abnormal distribution were expressed as the median with the interquartile range and Log10 transformed when analysed. According to the normality of the variables involved in the analysis, comparisons between two groups were conducted by a Student’s paired *t* test or sign-rank test, and relationships of inflammatory cytokines with metabolism parameters were estimated by Spearman or Pearson correlation coefficients. Categorical variables were analysed by the chi-square test. Spearman partial correlation analysis was used to adjust for confounding factors, including age, pregestational BMI, changes of BMI, gestational age, blood pressure, previous history of GDM, family history of diabetes and all the inflammatory cytokines. Results were considered statistically significant if two-tailed *P* < 0.05.

## Results

### Characteristics of subjects

The clinical characteristics of all participants have been described previously [14]. In brief, in addition to the increased blood pressure, fasting insulin, homeostasis model assessment of insulin resistance (HOMA-IR) and β-cell secretion (HOMA-β) in the GDM groups in the second trimester, GDM patients in both the second and third trimesters had higher previous history of GDM, family history of diabetes, HbA1c, fasting, 1-h and 2-h glucose levels and greater area under curve of glucose (AUCG) values than did the normal controls (all *P* < 0.05) (Table 1).

**Table 1:** Characteristics of the GDM and NGT groups in both the second and third trimesters

Characteristics	Participants in the second trimester			Participants in the third trimester		
	GDM (n = 200)	NGT (n = 200)	*P*	GDM (n = 130)	NGT (n = 130)	*P*
Age (years)	32 (29, 35)	32 (29, 35)	0.906	31 (29, 34)	31 (29, 33)	0.897
SBP (mmhg)	109.83 ± 10.14	106 (101, 113)	0.005			
DBP (mmhg)	67.44 ± .80	65 (61, 71)	0.022			
Pregestational BMI (kg/m^2^)	21.32 ± 2.59	21.26 ± 2.52	0.343	21.19 ± 2.29	21.06 ± 2.15	0.695
Normal weight, n (%)	171 (85.5)	173 (86.5)		112 (86.15)	118(90.77)	
Overweight, n (%)	24 (12)	23 (11.5)	0.93	18 (13.85)	12 (9.23)	0.24
Obesity, n (%)	5 (2.5)	4 (2)		0	0	
Changes of BMI (kg/m^2^)	2.69 ± 1.23	2.70 ± 1.23	0.792	5.15 ± 1.66	5.78 ± 1.35	0.001
Previous history of GDM, n (%)	19 (9.5)	1 (0.5)	< 0.001	12 (9.23)	3 (2.31)	0.017
Family history of diabetes, n (%)	48 (24)	5 (2.5)	< 0.001	37 (18.5)	23 (11.5)	0.039
Gestational age (weeks)	26 (25, 27)	25 (25, 26)	0.498	37 (36, 37)	36 (36, 37)	0.024
HbA1c (%)	5.20 (5.00, 5.40)	5.10 (5.00, 5.30)	0.003	5.40 (5.20, 5.60)	5.20 (5.00, 5.40)	< 0.001
FBG (mmol/L)	4.64 ± 0.53	4.36 ± 0.28	< 0.001	4.70 ± 0.54	4.37 ± 0.28	< 0.001
1h-PG (mmol/L)	9.88 ± 1.32	7.45 ± 1.24	< 0.001	9.71 ± 1.61	7.16 ± 1.43	< 0.001
2h-PG (mmol/L)	9.07 ± 1.24	6.66 ± 0.98	< 0.001	8.55 ± 1.42	6.34 ± 1.01	< 0.001
AUCG (mmol/L h)	16.74 ± 1.72	12.97 ± 1.56	< 0.001	16.33 ± 2.07	12.52 ± 1.77	< 0.001
Fasting insulin (mU/L)	6.79 (4.75, 10.78)	5.37 (3.73, 7.77)	0.001			
HOMA-IR	1.38 (0.95,2.21)	1.05 (0.71,1.53)	< 0.001			
HOMA-β	134.07 (88.68,206.35)	123.20 (81.80,215.58)	0.522			

Data are presented as the means ± SDs or median (interquartile range)

*SBP* systolic blood pressure, *DBP* diastolic blood pressure, *BMI* body mass index, *FBG* fasting blood glucose, *1h-PG* 1-h postprandial glucose, *2h-PG* 2-h postprandial glucose, *AUCG* area under curve of glucose from the 75-g OGTT, *HOMA-IR* homeostasis model assessment of insulin resistance, *HOMA-β* homeostasis model assessment index of β-cell secretion

### Inflammatory cytokine levels of the GDM and NGT groups in the second and third trimesters

**Fig. 1:**
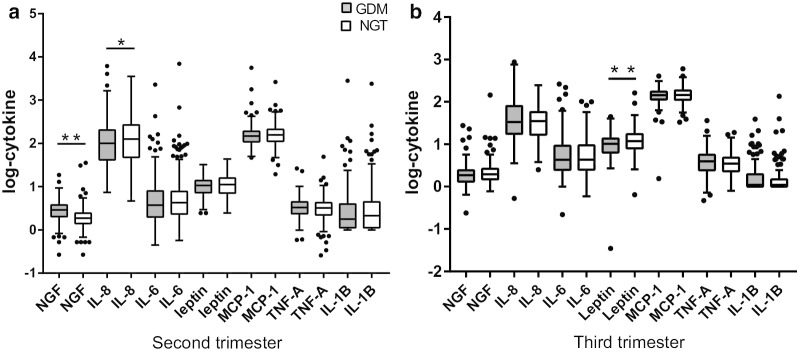
Serum levels of selected inflammatory cytokines in the second and third trimesters. The horizontal lines represent the medians, the tops and bottoms of the boxes represent quartiles, and the t-bars denote the 25th and 75th percentiles plus and minus the 1.5 interquartile distances. The solid circles represent outliers. *P < 0.05; **P < 0.001. *NGF* nerve growth factor, *IL-8* Interleukin-8, *IL-6* Interleukin-6, *MCP-1* monocyte chemoattractant protein 1, *TNF-α* tumor necrosis factor-alpha, *IL-β* Interleukin-1 beta

We compared the levels of the seven inflammatory factors between the GDM and NGT groups in both the second and third trimesters. The results showed that in the second trimester, serum NGF levels of GDM patients were remarkably higher than those of the normal controls (*P* < 0.001) (Table 2 and Fig. 1), whereas GDM patients had lower IL-8 levels (*P* = 0.015). However, in the third trimester, NGF and IL-8 no longer showed significant differences, while lower leptin levels were observed in the GDM group than in the NGT group (Table 2 and Fig. 1).

**Table 2:** Inflammatory cytokines in the second and third trimesters

	Subjects in the second trimester			subjects in the third trimester		
	GDM (n = 200)	NGT (n = 200)	*P*	GDM (n = 130)	NGT (n = 130)	*P*
NGF (pg/ml)	2.92 (2.05, 3.78)	1.87 (1.42, 2.43)	< 0.001	1.86 (1.33, 2.41)	1.97 (1.51, 2.64)	0.055
IL-6 (pg/ml)	3.69 (2.01, 7.82)	4.28 (2.36, 7.84)	0.217	4.32 (2.53, 9.21)	4.33 (2.53, 9.4)	0.838
Leptin (ng/ml)	10.74 (7.28, 13.79)	11.23 (7.08, 16.00)	0.099	10.33 (6.46, 13.53)	11.91 (7.94, 17.39)	0.031
IL-8 (pg/ml)	101.23 (42.03, 202.40)	126.46 (47.84, 268.77)	0.015	33.56 (17.58, 77.18)	34.89 (17.12, 57.27)	0.159
MCP-1 (pg/ml)	146.8 (109.86, 189.25)	158.37 (112.18, 211.55)	0.059	142.48 (114.32, 173.62)	145.73 (112.77, 185.09)	0.866
TNF-α (pg/ml)	3.33 (2.39, 4.44)	3.22 (2.23, 4.28)	0.432	3.94 (2.46, 5.59)	3.53 (2.28, 4.79)	0.121
IL-1β (pg/ml)	1.78 (1.13, 3.95)	2.15 (1.13, 4.45)	0.271	1.09 (1.01, 1.97)	1.09 (1.01, 1.49)	0.260

Data are median (interquartile range)

*GDM* gestational diabetes mellitus, *NGT* normal glucose tolerance, *NGF* nerve growth factor, *IL-6* Interleukin-6, *IL-8* Interleukin-8, *MCP-* monocyte chemoattractant protein 1, *TNFα* tumor necrosis factor-alpha, *IL-β* Interleukin-1beta

### Associations of selected inflammatory cytokines with glucose metabolism, insulin resistance and pancreatic β cell function

**Fig. 2:**
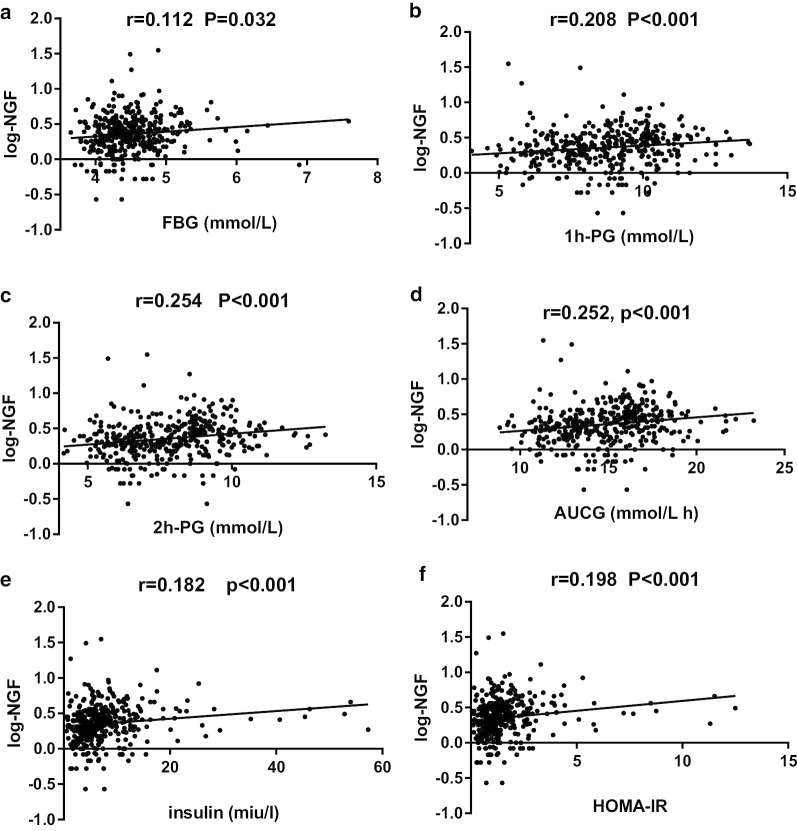
Correlations of NGF with glucose levels, insulin resistance and insulin secretion. r and p: adjusted for all the cytokines, maternal age, gestational age, pregestational BMI, changes of BMI, blood pressure, family history of diabetes and previous history of GDM. *NGF* nerve growth factor, *FBG* fasting blood glucose, *1h-PG* 1-h postprandial glucose, *2h-PG* 2-h postprandial glucose, *AUCG* area under curve of glucose from the 2-h OGTT, *HOMA-IR* homeostasis model assessment of insulin resistance, *HOMA-β* homeostasis model assessment index of β-cell secretion

**Fig. 3:**
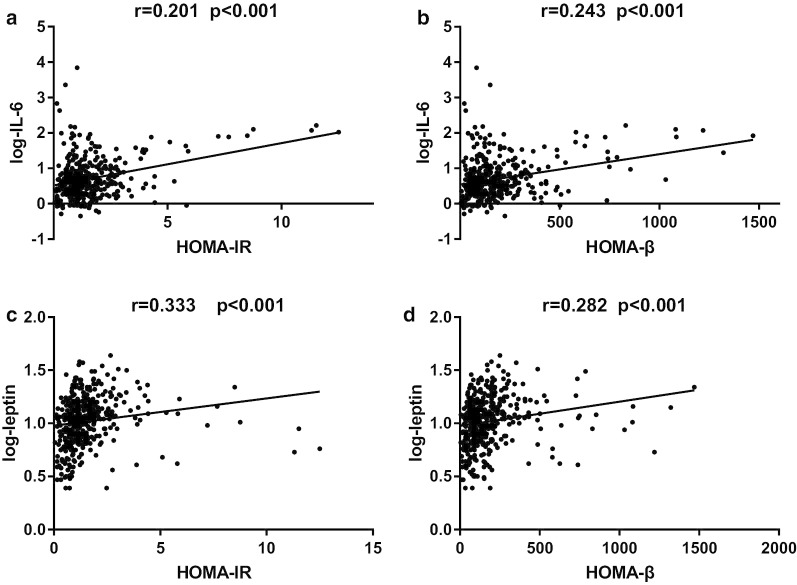
Correlations of IL-6 and leptin with insulin secretion and resistance. r and p: adjusted for all the cytokines, maternal age, gestational age, pregestational BMI, changes of BMI, blood pressure, family history of diabetes and previous history of GDM. *IL-6* Interleukin-6, *HOMA-IR* homeostasis model assessment of insulin resistance, *HOMA-β* homeostasis model assessment index of β-cell secretion

Because of the missing data related to glucose levels in the third trimester, we conducted correlation analysis for only the second trimester data. When we divided our samples into 2 groups according to inflammatory levels, we found higher NGF levels were associated with a higher risk of GDM even after adjusting for confounding factors (OR (95%CI) = 6.71 (4.02,11.18), *P* < 0.0001). Besides, the glucose levels, insulin levels, insulin resistance and secretion also increased with the increasing NGF levels (Additional file 1: Table S1). Next, we conducted correlation analysis, and the results showed that NGF was not only positively associated with fasting (*P* = 0.007), 1-h (*P* < 0.001) and 2-h glucose levels (*P* < 0.001) and AUCG values (*P* < 0.001), but also positively related to insulin resistance and pancreatic β cell function, as shown by fasting insulin (*P* < 0.001) HOMA-IR (*P* < 0.001) and HOMA-β (*P* = 0.043) (Fig. 2). The other inflammation-related factors, such as IL-6 and leptin, were positively correlated with only HOMA-IR and HOMA-β (all *P* < 0.001), and TNF-α was positively related to only HOMA-IR (*P* = 0.022) (Fig. 3). Except for the relationships between TNF-α and HOMA-IR and NGF and HOMA-β, the other relationships still existed even after adjusting for maternal age, pregestational BMI, gestational age, changes of BMI, blood pressure, family history of diabetes, previous history of GDM and all the other inflammatory cytokines (all *P* < 0.05). As women who were overweight and obese have a higher chance of being diabetic or having other metabolic dysfunction, we also conducted correlation analysis after excluding them from our study and reanalyzed the data. As expected, the new result showed the same trends just as we got above (Additional file 2: Table S2).

## Discussion

There have been many studies assessing the exact changes in inflammatory cytokines throughout normal gestation, but explorations of the relationships among these cytokines and metabolic indexes in Chinese pregnant women are still limited. Through our investigation, in addition to the positive associations of leptin, IL-8 and TNF-α with insulin resistance or secretion, NGF was found for the first time to be higher in GDM patients and positively associated with fasting and postprandial glucose metabolism, insulin resistance and pancreatic β cell function in the second trimester in Chinese.

NGF is an essential factor for the development of the nervous system in individuals and is necessary for healthy pregnancies [15, 16]. In addition, NGF is an inflammatory factor that can be induced by a variety of inflammatory diseases [17]. In many articles, NGF expression was shown to be increased in diabetes mellitus and it has been proven to be an important protective factor in diabetic neuropathy and vasculopathy [18–21]. We suspected that the vascular and neuroprotective effects of NGF also played significant roles in the physiological processes of GDM. In our study, serum levels of NGF were significantly higher in GDM patients than in normal controls, suggesting the essential role of NGF in GDM. However, the levels of NGF in this study were different from previous reports [22–24]. The difference of sample types might be part of the reasons as most of those studies were conducted with the plasm while we used the serum. In addition, the method we used which could detect all of seven inflammatory factors in one panel is also different from the traditional ELISA method.

Currently, studies have found that *NGF* and its receptor *Tyrosine kinase A (TrkA)* are expressed in many tissues, including β cells of the pancreas [25–27], and that high glucose levels could dramatically enhance the secretion of NGF and subsequently activate β-cell TrkA receptors to acutely promote glucose-stimulated insulin secretion [28, 29]. These results were consistent with our finding that NGF was positively related to glucose levels and insulin secretion in the second trimester. Moreover, NGF could reduce insulin resistance both in vitro and in vivo by activating insulin receptor substrate 1 (IRS1) [30, 31]. Therefore, NGF levels are supposed to have a negative relationship with insulin resistance. However, in our study, we found NGF levels were positively associated with insulin resistance, which might be ascribed to the compensatory adjustment. These data suggest that NGF acts as a protective factor in glucose metabolism and insulin sensitivity and plays a significant role in glucose-induced insulin secretion. Although the correlations between NGF and these metabolism indexes were poor as all r < 0.3, given the significant role of NGF in insulin signal pathway, and the close relationship between NGF and GDM, we could not neglect the essential role of NGF in GDM.

Compared to the increasing NGF levels and decreasing IL-8 levels in GDM patients in the second trimester, in the third trimester, only leptin levels showed a significant difference between the GDM group and NGT group. Considering the close relationships between glucose levels and inflammatory responses [32], we speculated that the different results might be due to the diverse glucose metabolism states of the participants, as 16.7% of the GDM patients in the third trimester had been treated with insulin. Unfortunately, there is no possibility to study the rest patients who didn't receive insulin therapy on account of the lack of data on glucose and insulin levels in the third trimester. Additionally, participants in the second and third trimesters were different, although they shared the same origin and showed no significant differences in age and pregestational BMI, we could not exclude the possibility of population heterogeneity.

Correlations of IL-6, leptin and TNF-α with diabetes have been explored extensively, and most of these studies obtained results similar to ours that TNF-α, IL-6 and leptin were positively associated with insulin resistance or secretion [33, 34]. However, after adjustments, the relationship between TNF-α and insulin resistance disappeared. In addition, no relationships were found between MCP-1 or IL-8 and glucose metabolism or insulin resistance and secretion, which was contradictory to the consequences of some studies [33, 35]. In this study, serum levels of IL-8 were statistical significance between GDM patients and normal controls but showed no relationships with glucose metabolism indexes or insulin secretion/resistance. However, we could not rule out the possibility that there were associations between IL-8 and other metabolism indexes causing the differences, although we did not have other clinical metabolism indexes to perform the correlation analysis. In addition, the special physiological status of GDM and the combined action of diverse diets, lifestyles and gene-profiles might partly account for these differences.

Our limitations are as follows: First, the study was a case–control study that could not explore the changes in serum inflammatory cytokine levels throughout the course of pregnancy. Moreover, in the third trimester, due to missing data on glucose levels and insulin secretion, no investigation of the relationships of inflammatory cytokines and glucose metabolism was conducted. Last, our participants in the second and third trimesters were from two different populations, and we could not determine if the differences in the second and third trimesters resulted from GDM therapy or population heterogeneity. Further longitudinal cohort and large sample size studies with more comprehensive data are still needed to explore which inflammation-related factors are involved in the progression of GDM throughout pregnancy.

## Conclusions

In conclusion, this systematic study investigated the relationships of selected inflammatory cytokines and GDM. We found that serum levels of NGF was higher in GDM patients in the second trimester, but not in the third trimester. Moreover, in the second trimester, in addition to the positive associations of IL-6 and leptin with insulin resistance, NGF was closely related to glucose metabolism, insulin resistance and pancreatic β cell function and might be a protective factor in the pathological process of GDM.

## Supplementary information

**Additional file 1: Table S1.** The relationships between inflammatory factors and clinical indexes.

**Additional file 2: Table S2.** Correlations of inflammatory factors and metabolism indexes after excluded women who were overweight or obese.

## Data Availability

The datasets analyzed during our current study are not publicly available but are available from the corresponding author on reasonable request.

## Abbreviations

AUCG: Area under curve of glucose from the 75-g oral glucose tolerance test
BMI: Body mass index
DBP: Diastolic blood pressure
FBG: Fasting blood glucose
GDM: Gestational diabetes mellitus
HOMA-IR: Homeostasis model assessment of insulin resistance
HOMA-β: Homeostasis model assessment index of β-cell secretion
IL-6: Interleukin-6
IL-8: Interleukin-8
IL-β: Interleukin-1 beta
MCP-1: Monocyte chemoattractant protein 1
NGT: Normal glucose tolerance
NGF: Nerve growth factor
OGTT: Oral glucose tolerance test
SBP: Systolic blood pressure
TNF-α: Tumor necrosis factor-alpha
1h-PG: 1-hour postprandial glucose
2h-PG: 2-hour postprandial glucose
