# Supplementary material for: Nerve growth factor is closely related to glucose metabolism, insulin sensitivity and insulin secretion in the second trimester: a case–control study in Chinese
Source: Nutr Metab (Lond). 2020 Nov 19;17:98. doi: 10.1186/s12986-020-00523-2 (PMC7678221; doi:10.1186/s12986-020-00523-2)
Supplement: Supplementary file 1 — Additional file 1: Table S1. The relationships between inflammatory factors and clinical indexes. [file 12986_2020_523_MOESM1_ESM.docx]

**Table S1. The relationships between inflammatory factors and clinical indexes**

|  | NGF | | | IL-6 | | |  | leptin |  |
| --- | --- | --- | --- | --- | --- | --- | --- | --- | --- |
| Characteristics | Group1  (<2.32 pg/ml) | Group 2  (>2.32pg/ml) | P | Group 1  (<3.89pg/ml) | Group 2  (>3.89pg/ml) | P | Group 1  (<10890pg/ml) | Group 2  (>10890pg/ml) | P |
| GDM patients | 59/200 | 141/200 | <0.001 | 111/200 | 89/200 | NS | 105/200 | 95/200 | NS |
| FBG (mmol/L) | 4.45±0.41 | 4.54±0.48 | 0.046 | NS | | | NS | | |
| 1h-PG (mmol/L) | 8.26±1.74 | 9.07±1.70 | <0.001 |  |  |  |  |  |  |
| 2h-PG (mmol/L) | 7.45±1.55 | 8.26±1.63 | <0.001 |  |  |  |  |  |  |
| AUCG (mmol/L h) | 14.22±2.43 | 15.48±2.41 | <0.001 |  |  |  |  |  |  |
| Fasting insulin (mU/L) | 3.78  (5.26, 7.51) | 4.65  (6.90, 10.07) | 0.001 | 3.78  (5.47, 8.24) | 4.19  (6.63, 10.59) | <0.001 | 3.38  (5.06, 7.16) | 4.91  (7.26, 11.05) | <0.001 |
| HOMA-IR | 0.73  (1.07, 1.50) | 0.90  (1.39, 2.00) | 0.001 | 0.75  (1.12, 1.66) | 0.89  (1.3, 2.03) | <0.001 | 0.70  (0.99, 1.41) | 1  (1.46, 2.09) | <0.001 |
| HOMA-β | 72.74  (117.49, 204.39) | 90.61  (135.02, 219.55) | <0.001 | 75.81  (113.21, 195.26) | 84.16  (144.13, 250.73) | <0.001 | 71.19  (107.97, 167.46) | 98.85  (165.82, 264.40) | <0.001 |

Data are presented as the means±SDs or median (interquartile range).

Abbreviations: NGF, nerve growth factor; IL-6, Interleukin-6; GDM, gestational diabetes mellitus; NS, no significance; FBG, fasting blood glucose; 1h-PG, 1-h postprandial glucose; 2h-PG, 2-h postprandial glucose; AUCG, area under curve of glucose from the 75-g OGTT; HOMA-IR, homeostasis model assessment of insulin resistance; HOMA-β, homeostasis model assessment index of β-cell secretion.
