# Supplementary material for: Nerve growth factor is closely related to glucose metabolism, insulin sensitivity and insulin secretion in the second trimester: a case–control study in Chinese
Source: Nutr Metab (Lond). 2020 Nov 19;17:98. doi: 10.1186/s12986-020-00523-2 (PMC7678221; doi:10.1186/s12986-020-00523-2)
Supplement: Supplementary file 2 — Additional file 2: Table S2. Correlations of inflammatory factors and metabolism indexes after excluded women who were overweight or obese. [file 12986_2020_523_MOESM2_ESM.docx]

**Table S2**

|  | NGF (pg/ml) | | | |  | IL-6 (pg/ml) | | | |  | Leptin (pg/ml) | | | |
| --- | --- | --- | --- | --- | --- | --- | --- | --- | --- | --- | --- | --- | --- | --- |
|  | r | P | r^a^ | P^a^ |  | r | P | r^a^ | P^a^ |  | r | P | r^a^ | P^a^ |
| 1h-PG (mmol/L) | 1.156 | 0.004 | 0.137 | 0.014 |  | NS | | NS | |  | NS | | NS | |
| 2h-PG (mmol/L) | 0.213 | <0.001 | 0.210 | <0.0001 |  | NS | | NS | |  | NS | | NS | |
| AUCG (mmol/L h) | 0.193 | <0.001 | 0.178 | 0.001 |  | NS | | NS | |  | NS | | NS | |
| HbA1c (%) | NS | | NS | |  | NS | | NS | |  | NS | | NS | |
| Fasting insulin (mU/L) | 0.174 | 0.001 | 0.169 | 0.002 |  | NS | | NS | |  | NS | | NS | |
| HOMA-IR | 0.184 | 0.001 | 0.174 | 0.002 |  | 0.329 | <0.001 | 0.441 | <0.001 |  | 0.193 | <0.000 | 0.148 | 0.008 |
| HOMA-β | NS | | 0.110 | 0.049 |  | 0.309 | <0.002 | 0.441 | <0.002 |  | 0.200 | <0.001 | 0.166 | 0.003 |

**Correlations of inflammatory factors and metabolism indexes after excluded women who were overweight or obese**

a: adjusted for all the cytokines, maternal age, gestational age, pregestational BMI, changes of BMI, blood pressure, family history of diabetes and previous history of GDM.

Abbreviations: NGF, nerve growth factor; IL-6, Interleukin-6; 1h-PG, 1-h postprandial glucose; 2h-PG, 2-h postprandial glucose; AUCG, area under curve of glucose from the 75-g OGTT; HOMA-IR, homeostasis model assessment of insulin resistance; HOMA-β, homeostasis model assessment index of β-cell secretion; NS: no significance.
